# An Asian traditional herbal complex containing *Houttuynia cordata* Thunb, *Perilla frutescens* Var. *acuta* and green tea stimulates hair growth in mice

**DOI:** 10.1186/s12906-017-2003-x

**Published:** 2017-12-02

**Authors:** Mun Su Chung, Woong Jin Bae, Sae Woong Choi, Kyu Won Lee, Hyun Cheoul Jeong, Fahad Bashraheel, Seung Hwan Jeon, Jin Woo Jung, Byung Il Yoon, Eun Bi Kwon, Hyun A Oh, Sung Yeoun Hwang, Sae Woong Kim

**Affiliations:** 1 Department of Urology, Catholic Kwandong University, International St. Mary’s Hospital, Incheon, Republic of Korea; 2 Catholic Integrative Medicine Research Institute, College of Medicine, The Catholic University of Korea, Seoul, Republic of Korea; 3 Department of Urology, College of Medicine, The Catholic University of Korea, Seoul St. Mary’s Hospital, 222, Banpo-daero, Seocho-gu, Seoul, 06591 Republic of Korea; 4 Department of Research and development, Korea Bio Medical Science Institute, Seoul, Republic of Korea

**Keywords:** Hair regeneration, C57BL/6, *Houttuynia cordata*, *Perilla frutescens* Var. *acuta*, Green tea

## Abstract

**Background:**

*Houttuynia cordata* Thunb (HC) is a traditional herbal medicine widely used in Asia for the treatment of patients with alopecia, usually in combination with other two herbal medicines (*Perilla frutescens* var. *acuta* (PFVA) and green tea (GT)). However, the effect of this herbal complex has not been clearly demonstrated. We sought to determine the hair growth-promoting effect of this herbal complex (HC, PFVA, and GT) in the animal model.

**Methods:**

Six-week-old male C57BL/6 mice were randomly divided into four groups (negative control, finasteride (1 mg/kg) as a positive control, and two (200 and 400 mg/kg) concentrations of the herbal complex as experimental groups) and were fed its corresponding medications orally for 25 days. Hair growth was evaluated visually and microscopically. Western blot analysis for insulin-like growth factor (IGF)-1 and transforming growth factor (TGF)-*β*1 was performed.

**Results:**

The herbal complex exhibited hair growth-promoting activity in C57BL/6 mice. Grossly, the area of hair regrowth was 55.1 (±3.8) %, 70.2 (±6.3) % and 83.5 (±5.7) % in negative control, herbal complex 200 mg/kg and 400 mg/kg group, respectively. In histologic examination, the hair follicle count in deep subcutis was 2.6 (±0.7), 5.8 (±0.7) and 8.6 (±1.2) and the diameter of hair follicles was 11.9 (±5.0) μm, 17.4 (±3.9) μm and 22.8 (±5.2) μm in negative control, herbal complex 200 and 400 mg/kg group, respectively. The expression of IGF-1 was 0.14 (±0.01), 0.23 (±0.02) and 0.24 (±0.01) and the expression of TGF-*β*1 was 0.26 (±0.01), 0.19 (±0.02) and 0.15 (±0.01) in negative control, the 200 and 400 mg/kg group, respectively.

**Conclusions:**

This data provides adequate preliminary experimental evidence to support the hair regeneration effect of this herbal complex.

**Electronic supplementary material:**

The online version of this article (10.1186/s12906-017-2003-x) contains supplementary material, which is available to authorized users.

## Background

There are currently only two FDA-approved drugs for treatment of androgenetic alopecia, oral finasteride and a topical minoxidil [1]. Finasteride, a competitive inhibitor of type II 5*α*-reductase, inhibits the conversion of testosterone to dihydrotestosterone and, thereby decreases androgen-dependent miniaturization of hair follicles in the scalp [2]. However, this drug is contraindicated in fertile or pregnant women due to its potential risk of malformation of the external genitalia in male fetuses. Minoxidil, a potassium channel opener, is known to stimulate hair growth, although its mechanism of action is not yet fully understood. However, the effect of this drug is variable and temporary, and several adverse effects, such as pruritus, dermatitis, and irritation have been reported [3, 4]. Consequently, there is still an unmet need for the development of novel, ideal hair growth promoters with minimal adverse effects. Currently, numerous drugs including folk medications are being used, although evidence of their efficacy and adverse effects are unclear and still under investigation [5–11]. The possible mechanisms of action of the current folk medications include dermal papillae activation, enhanced blood supply in the perifollicular area, anti-inflammatory action, inhibition of 5-alpha-reductase, and nutritional support for hair follicles [12].

*Houttuynia cordata Thunb* (HC), which is widely known as *Eu-Sung-Cho* in Korea and China, is a perennial herbaceous plant from the Saururaceae family and is widely grown in southeast Asia (in Korea, China, Japan, Taiwan, and India) [13, 14]. For decades, the root, young shoots, leaves of HC or the whole plant have been used as a traditional folk medicine for various medicinal purposes throughout southeast Asia [15–18]. For example, in India, its leaf juice was used orally to treat cholera, dysentery, and for purification of blood [16]. It was also used as an antidote and astringent [17]. HC was used orally to treat various minor illnesses, such as coughs, swelling, enteritis and fever in decoction form [15]. Topically, it was used to treat snake bites and skin disorders. In the Indo-China region, the entire plant was used orally for cooling, treating furuncle, and inducing menstruation. Similarly, in Korea and China, the leaves were traditionally used orally to treat dysentery, gonorrhea, measles, hemorrhoids, and eye trouble. It was also used to drain pus and to enhance voiding [18]. The history of such effects originated from an oriental pharmaceutical text, *Ben cao gang mu* [19] (first published in 1593, Ming Dynasty of China), which is regarded as the most complete and comprehensive medical book ever written in the history of traditional Chinese medicine.

Subsequently, since the 1990’s, experimental evidence to support the efficacy of HC has accumulated in various biological fields. Numerous studies [13, 15] have proven that HC exhibits anti-inflammatory [15], anti-viral [20], anti-bacterial, anti-cancer [21], and anti-allergy [22] activities.

Regarding its bioactive components, phytochemical investigations on HC conducted up through 2012 reported numerous phytoconstituents of this plant. Various types of chemical constituents, such as flavonoids (quercetin, isoquercitrin, rutin), aristolactams, 5,4-dioxoaporphines, oxoaporphines, amides, indoles, ionones, polyphenol (including procyanidin B-2, catechin), benzenoids, steroids and different volatile oils, have been isolated from HC [15, 23–25].

Besides the above discussed biological effects, HC is traditionally used orally to treat patients with alopecia. This practice also originated from the recommendations in *Ben cao gang mu* [19]. Although experimental evidence regarding its hair regeneration effect has not yet been established, HC has been popularly used as a herbal hair-promoter owing to its anecdotal efficacy in Asian countries, especially in Korea. In addition, HC is often used in combination with other two herbal medicines (*Perilla frutescens* Britton var. *acuta* (PFVA) and green tea (GT)). Therefore, this “tri-mix” herbal complex (HC, PFVA, and GT) is commercialized and is being widely used by alopetic patients in Korea.

PFVA (called as *Ja-So-Yup*) is a naturalized edible plant, and is distributed widely throughout the Himalayan mountains and East and Southeast Asia, especially in Korea and Japan. Traditionally, it has been orally used to treat all kinds of diseases such as cold, fever, chills, headache, stuffy nose, cough or chest discomfort. PFVA extract has been reported to contain numerous functional compounds with anti-allergenic, anti-inflammatory, anti-microbial, and anti-bacterial effects [26–29].

GT (called as *Nok-Cha*) is one of the most prevalent drinks in Asia. Traditionally, it has been consumed (as beverage) by East Asian people for health promotion and is known to have anti-cancer and anti-oxidant actions, primarily mediated by *epigallocatechin-3-gallate* (EGCG) [30]. With respect to androgen metabolism, EGCG inhibits 5*α*-reductase [31] and represses the expression of androgen receptor genes [32], and thereby exhibits a potential action for the prevention or treatment of androgen-dependent disorders. Kwon et al. [33] reported that EGCG stimulates human hair growth via proliferative and anti-apoptotic effects on dermal papilla cells. Similarly, topical EGCG was reported to reduce testosterone-induced apoptosis of hair follicles in a mouse model.

Among various species of animals, the mouse model is most widely reported for hair growth promotion studies due to availability of large data base and specific mutants such as nude, hairless, rhino, etc. The periodic intervals of rodent hair cycles, particularly the duration of the anagen phase are much more consistent and less susceptible to iatrogenic influences. The disadvantages associated with the mouse model include a high follicle density and the fact that the rodent hair cycle progresses in a wave pattern that sweep posterior and dorsally [34], unlike the mosaic pattern seen in humans [35]. The truncal pigmentation of C57/BL6 mice is entirely dependent on their follicular melanocytes. The truncal epidermis in this species lacks melanin-producing melanocytes and melanin production is strictly coupled to anagen phase of hair growth. Thus, the strict coupling of follicular melanogenesis and hair follicle cycling leads to characteristic changes in skin pigmentation during anagen development. For these reasons, pigmented C57/BL6 mice are the most commonly used strain for hair growth studies.

In this study, we aimed to evaluate the hair promoting effect of the herbal complex (a combination of HC, PFVA, and GT) using C57BL/6 mice. To the best of our knowledge, this is the first experimental study on this herbal medication for treatment of hair loss.

## Methods

### Preparations of HC, PFVA and GT extracts and herbal complex

The major ingredients in the herbal formula were obtained from three plants (HC, PFVA and GT). HC and PFVA were collected at Yeongcheon, Gyongsangbuk-do, Republic of Korea and GT at Boseong, Jeollanam-do, Republic of Korea. Each of these plants was identified by Dr. Sung Yeoun Hwang. Voucher specimens were deposited in a publicly available herbarium, Globalherb Inc. (Korea excellent medicinal herbs distribution support center; Andong, Gyongsangbuk-do, Republic of Korea). Distilled water extracts: Each component (50 kg + 500 L water) was boiled at 96–100 °C for 4 h. The extract was filtered, and the water from the filtrate was removed by a pressure dried. The HC, PFVA and GT was mixed in a proportion of 2 (HC): 1 (PFVA): 1 (GT) of extracts by weight. The extract powder was then dissolved in D.W. or dimethyl sulfoxide and filtered (pore size, 0.22 μm), then kept at 4 °C for use. Korea Bio Medical Science Institute Co. Ltd., a venture company that develops oriental herbal medicines, produced this product (MGD-100) as a health supplement.

### HPLC analysis of each herbal extract component

**Fig. 1:**
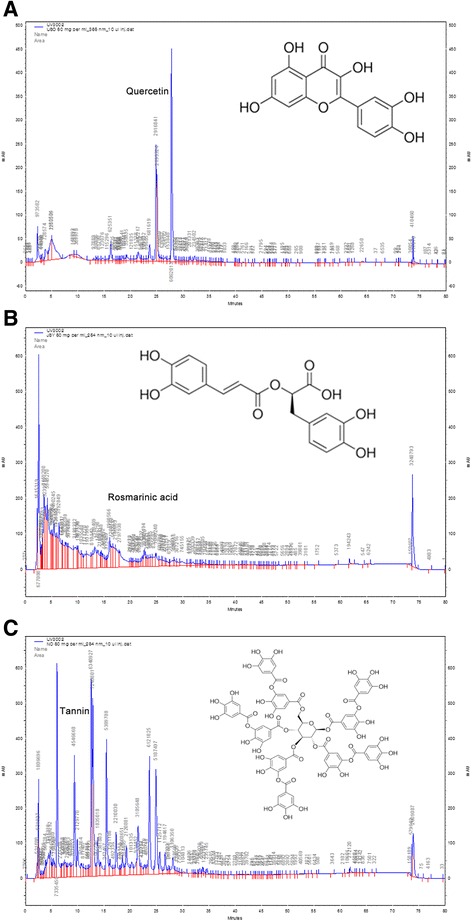
HPLC analysis of each herbal extract component. Chromatographic conditions: SHISEIDO Capcallpak MGII C18 column (4.6 × 250 mm, 5 μm). **a** HPLC profile of HC at the wavelength of 365 nm. The mobile phase consisted of 0.1% Phosphoric acid in DW (**a**) and methanol (**b**), using a gradient elution of 30% B at 0–5 min, 30–90% B at 5–25 min, 90% B at 25–33 min, 90–30% B 33–33.2 min, and 30% B at 33.2–40 min. The solvent flow-rate was 1.0 mL/min and the column temperature was 40 °C. **b** HPLC profile of PFVA at the wavelength of 254 nm*.* The mobile phase consisted of 0.05% Phosphoric acid in DW (**a**) and acetonitrile (**b**), using a gradient elution of 5–100% B at 0–10 min, 100% B at 10–13 min, and 100–5% B at 13–20 min. The solvent flow-rate was 1.0 mL/min and the column temperature was 35 °C. **c** HPLC profile of GT at the wavelength of 254 nm. The mobile phase consisted of 0.05% Acetic acid in DW (**a**) and methanol (**b**), using a gradient elution of 5–100% B at 0–10 min, 100% B at 10–15 min, and 100–5% B at 15–20 min. The solvent flow-rate was 1.0 mL/min and the column temperature was 35 °C. The contents of quercetin, RA and tannin were determined as 0.14 ± 0.08 mg/g of HC, 17.7 ± 0.80 mg/g of PFVA and 56.74 ± 0.17 mg/g of GT, respectively. HPLC, High performance liquid chromatography; HC, *Houttuynia cordata* Thunb; PFVA, *Perilla frutescens* Britton *var. acuta*; GT, green tea; RA, rosmarinic acid

High performance liquid chromatography (HPLC)-UV analyses (Fig. 1) were performed using an SHISEIDO SP3202 pump equipped with a SP3002 UV detector (SHISEIDO, Ginza, Tokyo, Japan); the separation was performed on a SHISEIDO Capcallpak MGII C18 column (4.6 × 250 mm, 5 μm). As a reference standard, we used quercetin, rosmarinic acid (RA) and tannin for HC, PFVA and GT, respectively. Quercetin: The mobile phase consisted of 0.1% Phosphoric acid in DW (A) and methanol (B), using a gradient elution of 30% B at 0–5 min, 30–90% B at 5–25 min, 90% B at 25–33 min, 90–30% B 33–33.2 min, and 30% B at 33.2–40 min. The solvent flow-rate was 1.0 mL/min and the column temperature was 40 °C. The detection wavelength was set at 365 nm. RA: The mobile phase consisted of 0.05% Phosphoric acid in DW (A) and acetonitrile (B), using a gradient elution of 5–100% B at 0–10 min, 100% B at 10–13 min, and 100–5% B at 13–20 min. The solvent flow-rate was 1.0 mL/min and the column temperature was 35 °C. The detection wavelength was set at 254 nm. Tannin: The mobile phase consisted of 0.05% Acetic acid in DW (A) and methanol (B), using a gradient elution of 5–100% B at 0–10 min, 100% B at 10–15 min, and 100–5% B at 15–20 min. The solvent flow-rate was 1.0 mL/min and the column temperature was 35 °C. The detection wavelength was set at 254 nm.

### Animals

Six-week-old male C57BL/6 mice (*N* = 20, purchased from OrientBio, Korea) were allowed to adapt to their new environment for one week, with food and water provided ad libitum. Animals were housed in polypropylene cages maintained under standard conditions of a 12 h light and dark cycle, 24 ± 0.5 °C and 55 ± 5% humidity. Body weights were recorded weekly. After acclimatization for 7 days, we anesthetized all mice with an intraperitoneal injection of a mixture of rumpun 15 mg/kg (Bayer, Leverkusen, Germany) and zoletil 50 mg/kg (Virbac, Carros, France). The dorsal hair (approximately 2 cm in width and 6 cm in length) of seven-week-old C57BL/6 mice, with all of the HFs in the telogen phase, were shaved using animal clippers to synchronize hair follicle growth. Further hair was eliminated using hair removal cream (Oxy Reckitt Benckiser, Seoul, Korea). The experimental protocol was approved by the Institutional Animal Care and Use Committee in the School of Medicine, The Catholic University of Korea (CUMC-2015-0198-01).

The mice were randomly divided into four groups according to the following treatments: distilled water as a vehicle as the negative control group (*N* = 5), finasteride (1 mg/kg) as a positive control group (N = 5), herbal complex = 2:1:1200 mg/kg group (N = 5), and herbal complex = 2:1:1400 mg/kg group (N = 5), as experimental groups. Each mouse received its corresponding medications for 25 days, starting 1 day after depilation. Herbal complex and finasteride were dissolved in 1.0 mL of distilled water and administered orally through an 8 F red Rob-Nel catheter once a day. Dose translation of our herbal complex (from human dose to animal dose) was determined using a formula [36], which is based on body surface area and Km factor (Km = 37 for human and Km = 3 for mouse): Human equivalent dose (mg/kg) = Animal dose (mg/kg) multiplied by [Animal Km/Human Km]. The human dose of this herbal complex was determined based on the recommendation of *Ben Cao Gang Mu.*

To evaluate safety of the herbal complex, the condition of each mouse was observed daily to detect abnormalities such as changes in physical appearance, behavioral signs and food intake throughout the study period. At day 25, euthanasia was performed by isoflurane followed by CO2 (Isoflurane was provided from a vaporizer at 5% with oxygen flow rate at 1 L/min. Mice were monitored continuously during the procedure, and once the mouse was immobile (except for breathing) for 1 min, compressed CO2 gas was provided at 100% chamber volume per minute).

### Gross morphology and histologic analysis

The back skin of mice was observed and photographed with a digital camera at day 0, 10, and 25 after depilation. After sending the image to a computer, we measured the area according to the following formula: [% Hair regrowth = hairy black area ÷ hair removal area] to quantitatively compare the degree of hair regrowth. The boundary between black and gray areas of skin was defined subjectively, by a single doctor (MSC). The measurements were done blinded to treatment. The skin was then isolated to examine the histological features after the sacrifice of all mice at day 25. Individual skin samples were fixed in 10% paraformaldehyde (Sigma–Aldrich, USA), and the tissues were then dehydrated using alcohol and xylene and embedded in paraffin using an automatic tissue processor, sectioned to 4 μm thickness with a microtome, and stained with hematoxylin and eosin. Then, histological analyses were performed using light microscopy (Olympus BX50; Olympus Tokyo, Japan). Using a digital photomicrograph, we counted the number of hair follicles and all of the images were cropped in a fixed area (150 pixels in width). Data were evaluated from representative areas at a fixed magnification of 100×. Hair follicle mean diameter, distance between hair follicle and dermis, and length of hair follicle were measured under the microscope.

### Western blot analysis

We analyzed the expression of insulin-like growth factor (IGF)-1 (a stimulator of hair growth) and transforming growth factor (TGF)-*β*1 (an inhibitor of hair growth) in the four groups of experimental mice. The levels of IGF-1 and TGF-*β*1 protein in skin were measured by Western blotting. Fifty milligrams of tissue with lysis buffer (50 mM Tris-HCl, 120 mM NaCl, 2 nM EDTA, 1 mM EGTA, 1% Triton X-100) was homogenized (Tissue Tearor, Biospec, Korea) and the homogenized samples (50 μg of protein) were subjected to 15% SDS-PAGE and then transferred to a PVDF membrane. Membranes were blocked in 5% skim milk for 1 h, incubated with primary antibodies (IGF-1, TGF-*β*1, Abcam, Cambridge, UK) overnight (4 °C) at appropriate dilutions (1:1000), and then incubated with the secondary antibody (goat anti-rabbit IgG, Stressgen, USA) conjugates for 1 h at room temperature. Blots were developed using a commercial enhanced chemiluminescence system, and densitometric analysis was performed using the Image Analyzer system (Syngene, UK).

### Statistical analysis

Results are presented as mean ± standard deviation (SD). Data were analyzed by one-way analysis of variance (ANOVA) followed by Duncan’s multiple comparison test. We used SPSS, version 18 (SPSS Inc., Chicago, IL, USA) for all statistical analyses. A value of *p* < 0.05 was considered statistically significant.

## Results

### The preparation of herbal extracts

The yield of the obtained HC, PFVA and GT as calculated from dried raw material was 13%, 9% and 11%, respectively. From the HPLC chromatogram, the contents of quercetin, RA and tannin were determined as 0.14 ± 0.08 mg/g of HC, 17.7 ± 0.80 mg/g of PFVA and 56.74 ± 0.17 mg/g of GT, respectively. The HPLC profile of HC, PFVA and GT (at the wavelength of 365 nm, 254 nm and 254 nm, respectively) is shown in Fig. 1.

### Effects of the herbal complex on hair growth in mice

**Fig. 2:**
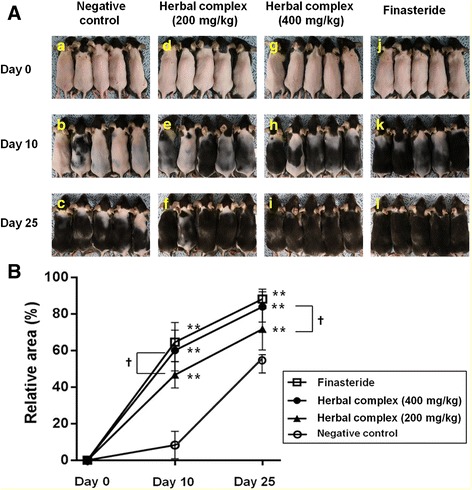
Gross observation and measurement of the area of hair regrowth in C57BL/6 mice. **a** After the dorsal skin was shaved, each mouse were orally administered the distilled water (negative control group; **A-C**), herbal complex = 2:1:1200 mg/kg (**D-F**) or herbal complex = 2:1:1400 mg/kg (**G-I**) group, and finasteride (1 mg/kg, as positive control group; **J-L**) for 25 days. Herbal complex and finasteride were dissolved in 1.0 mL of distilled water and administered through an 8 F red Rob-Nel catheter once a day. Back skin was photographed at day 0, 10, and 25 after depilation. As the duration of treatment became prolonged, hair growth was more readily visible. The experimental group (both the 200 and 400 mg/kg groups) showed darker skin than the negative control group. **b** The area of hair regrowth by image software. Data are presented as the mean ± SD. **p < 0.001 compared with negative control, †*p* < 0.05 herbal complex 200 mg/kg vs. 400 mg/kg group. Herbal complex = *Houttuynia cordata* Thunb, *Perilla frutescens* Britton var. acuta, and Green tea

Back skin was photographed at day 0, 10, and 25 after depilation. Until day 5 after depilation, we noted little change in all groups. As the duration of treatment became prolonged, hair growth was more readily visible. The experimental group (both the 200 and 400 mg/kg groups) and the positive control finasteride group showed darker skin than the negative control group (Fig. 2a). When we measured the area of hair regrowth by image software, much higher regrowth (*p* < 0.001) was noted in all experimental groups and in the finasteride group, relative to the negative control group (Fig. 2b). Higher dose (400 mg/kg) in the experimental group resulted in higher regrowth of hair than observed in the low dose (200 mg/kg) group (Fig. 2b).

### Histologic examination of the observed mouse hair growth

**Fig. 3:**
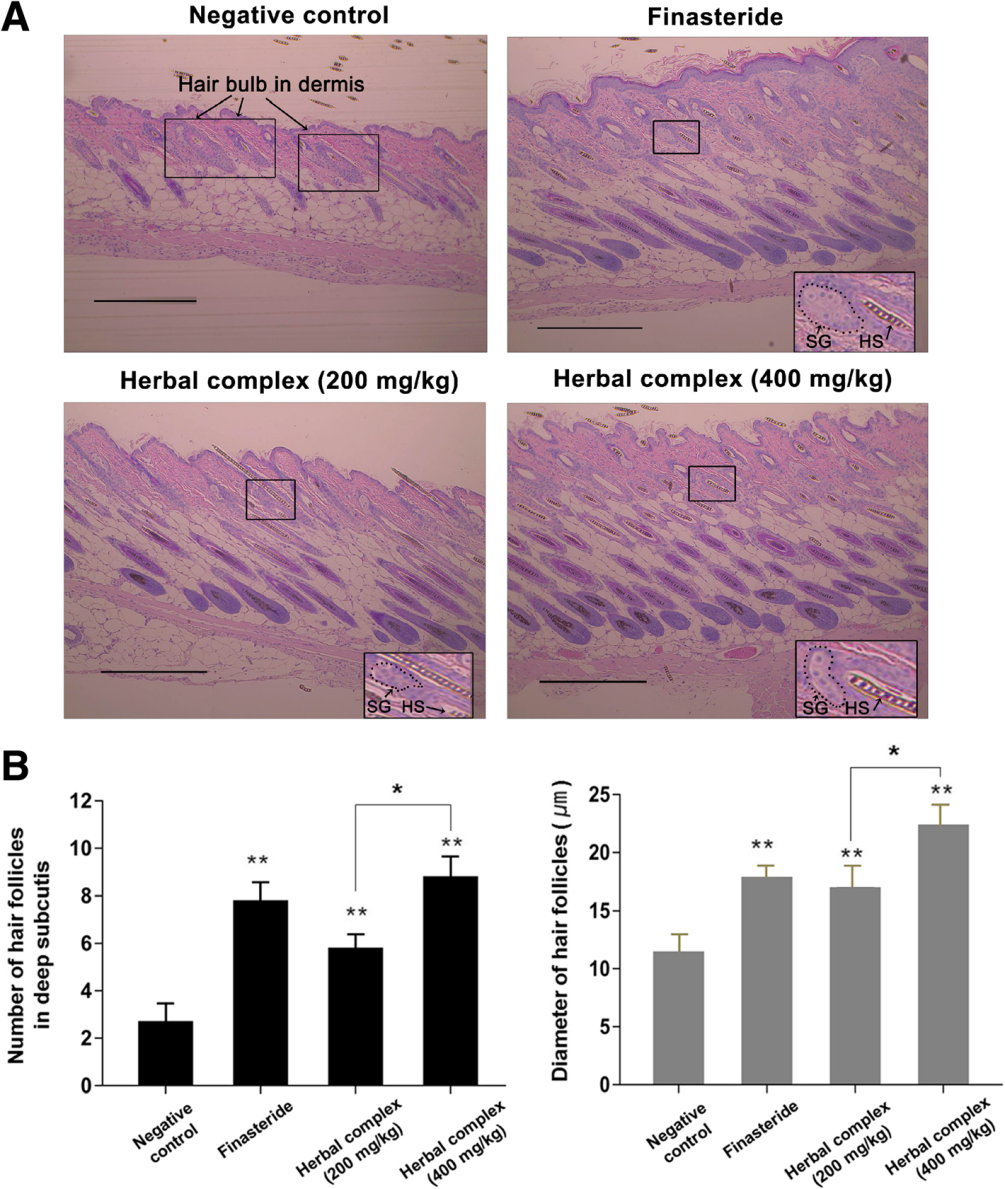
Histologic analysis of hair follicle growth in C57BL/6 mice. Skin samples were fixed in 10% paraformaldehyde, embedded in paraffin, sectioned, and stained with hematoxylin and eosin. **a** Hairs in the negative control group on day 25 were in the early anagen phase (hair bulb in the dermis). In contrast, the hair follicles in the experimental groups and in the finasteride group were at least in the anagen IIIc-IV phase, showing maximal size of hair bulb, hair follicle deep in the subcutis, and newly formed hair shaft reaching the level just below the sebaceous gland. The higher dose (400 mg/kg) used in the experimental group resulted in deeper hair bulb and larger hair follicle than observed in the low dose (200 mg/kg) group. (×100 objective magnification, scale bar = 100 μm). **b** The hair follicle counts in deep subcutis and the diameter of hair follicles. Data are presented as the mean ± SD. **p < 0.001 compared with negative control, *p < 0.05 herbal complex 200 mg/kg vs. 400 mg/kg group. Herbal complex = *Houttuynia cordata* Thunb, *Perilla frutescens* Britton var. acuta, and Green tea. SG, sebaceous gland; HS, hair shaft

As shown in Fig. 3, we observed more hair follicles in deep subcutis in all experimental groups and in the finasteride group compared with the negative control group, in the histologic analysis conducted at day 25 after depilation. We analyzed the histologic findings using stage-specific characteristics of hair follicle, based on the accepted morphological guidelines [37] which describe six main stages and three substages of follicular growth (anagen), eight stages of follicular regression (catagen) and the quiescence stage (telogen) of the hair cycle, modified from previous classifications [38–40]. Hairs in the negative control group on day 25 were in the early anagen phase, in which the bulb in the dermis and enlarged dermal papilla are distinct characteristics. In contrast, the hair follicles in the experimental groups and in the finasteride group were at least in the anagen IIIc-IV phase, showing maximal size of hair bulb, thinner dermal papilla, hair follicle deep in the subcutis, and newly formed hair shaft reaching the level just below the sebaceous gland. The higher dose (400 mg/kg) used in the experimental group resulted in deeper hair bulb and larger hair follicle than observed in the low dose (200 mg/kg) group (*p* < 0.05; Table 1 and Fig. 3b).

**Table 1:** Effect of the herbal complex: histologic findings at day 25

	Negative	Finasteride	Herbal complex (200 mg/kg)	Herbal complex (400 mg/kg)
Mean (**±**SD)				
Diameter of hair follicle (μm)	11.9 (**±**5.0)	18.3 (**±**5.2)	17.4 (**±**3.9)	22.8 (**±**5.2)
Distance (μm) between hair bulb and dermis	10.3 (**±**5.3)	94.4 (**±**8.9)	87.7 (**±**14.0)	109.2 (**±**11.5)
Length of hair follicle (μm)	93.1 (**±**34.8)	246.7 (**±**25.2)	217.7 (**±**22.7)	270.8 (**±**26.8)

*SD* standard deviation

### Expression of IGF-1 and TGF-*β*1 in the treated mice

**Fig. 4:**
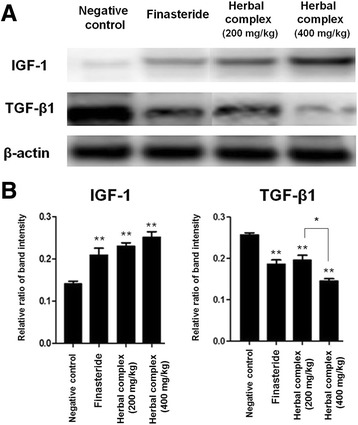
IGF-1 and TGF-*β*1 Western blot analysis. **a** A western blot assay was performed to compare each group. In the experimental group (both the 200 and 400 mg/kg groups), IGF-1 expression was much higher and TGF-*β*1 expression was much lower compared with the negative control group (*p* < 0.05). Higher doses (400 mg/kg) in the herbal complex treated animals resulted in decreased expression of TGF-*β*1 (*p* < 0.05) than seen in the low-dose (200 mg/kg) groups. **b** Quantitative representation of IGF-1 and TGF-*β*1 expression. Data are presented as the mean ± SD. **p < 0.001 compared with negative control, *p < 0.05 herbal complex 200 mg/kg vs. 400 mg/kg group. Herbal complex = *Houttuynia cordata* Thunb, *Perilla frutescens* Britton var. acuta, and Green tea

IGF-1 expression was higher in all experimental groups and in the positive control group relative to the negative control group (*p* < 0.05). The expression of IGF-1 was increased by 64% and 70% in experimental groups compared to negative control (in the 200 and 400 mg/kg group, respectively). TGF-*β*1 expression was lower in all experimental groups and the positive control group compared with the negative control group (*p* < 0.05). The expression of TGF-*β*1 was decreased by 24 and 44% in experimental groups (the 200 and 400 mg/kg groups, respectively) compared to negative control group. The higher doses (400 mg/kg) used in the experimental group resulted in lower expression of TGF-*β*1 (*p* < 0.05) relative to the low dose (200 mg/kg) groups (Fig.4).

## Discussion

In this study, the tri-mix herbal complex (HC, PFVA, and GT) exhibited hair growth-promoting activity based on analysis of gross morphology, histological finding, and expression of IGF-1 and TGF-*β*1 in a C57BL/6 mice model.

IGF-1 has been reported to increase the growth of cultured hair follicles. Numerous studies have reported that IGF-1 enhances follicular proliferation, tissue remodeling, and the hair growth cycle, as well as follicular differentiation [41–44]. In addition, it is well known that IGF-1 might be associated with an increased risk of cancer [45]. However, there has been no report that the herbal medicines (HC, PFVA and GT) have any association with carcinogenesis. TGF-*β*1, which plays an important role in the regulation of differentiation, proliferation, and apoptosis in various types of cells, is a possible negative regulator of hair follicle growth and is highly expressed during the catagen and telogen phases [46]. Consequently, IGF-1 and TGF-*β*1 are considered to stimulate and inhibit hair growth, respectively.

Regarding the undiscovered mechanism of action of the hair promoting effect of HC, we believed that HC has a certain element that modulates IGF-1 and TGF-*β*1: we found a report that procyanidin B-2, which is one of the principle element of HC [12, 15], can inhibit TGF-*β*1 (leading to conversion of telogen follicles to anagen hair follicles) [12]. In 2000, there was a report that topical application of procyanidin B resulted in an increased total hair count (6.68/0.25 cm^2^ in procyanidin B group vs. 0.08/0.25 cm^2^ in placebo group, after sequential use for 6 months) in male patients [47]. Accordingly, a possible explanation may be that some elements (such as procyanidin B) in HC modulate IGF-1 and TGF-*β*1, resulting in the induction of anagen hair regrowth. Consequently, we analyzed the expression of IGF-1 and TGF-*β*1 after HC-containing herbal medicine treatment. As shown in Fig. 4, IGF-1 expression was much higher (*p* < 0.05) and TGF-*β*1 expression was much lower (*p* < 0.05) in the experimental group (both the 200 and 400 mg/kg groups) compared with the negative control group (The expression of IGF-1 was 0.14 (±0.01), 0.23 (±0.02) and 0.24 (±0.01) and the expression of TGF-*β*1 was 0.26 (±0.01), 0.19 (±0.02) and 0.15 (±0.01) in negative control, the 200 and 400 mg/kg group, respectively). We think our results may suggest the mechanism of action of HC with respect to hair regeneration.

HPLC–UV chromatogram in current study showed that quercetin was contained in HC. Quercetin is one of the most abundantly distributed flavonoids found in various food sources such as apples, cranberries, green leafy vegetables, seeds, flowers, olive oil, etc. [48]. This flavonoid has been reported to have anticarcinogenic and antiinflammatory properties and have therapeutic potentials in hypertension and neurodegenerative diseases [48, 49]. In terms of hair loss, Wikramanayake et al. [50] reported the positive effect of quercetin for alopecia areata in the C3H/HeJ model. The authors demonstrated that the effect of quercetin on hair loss was associated with reduced heat shock protein 70 and that quercetin had potential to counter the breakdown of the immune privilege in alopecia areata. However, there are few reports on the benefit of quercetin for treatment of androgenetic alopecia. Further studies are needed to address this.

RA is a polyphenolic phytochemical found in several medicinal plants of the Lamiaceae family, such as rosemary (*Rosmarinus officinalis* L.), spearmint (*Mentha* spp.), and lemon balm (*Melissa officinalis* L.), and also in plants used in traditional Chinese medicine, such as *Perilla frutescens* (L.) Britton, *Salvia miltiorrhiza* Bunge, and *Rabdosia rubescens* (Hemsl.) *H. Hara* [51]. RA has been reported to have adstringent, antioxidative, antiinflammatory, antimutagen, antibacterial and antiviral activities [51, 52]. Regarding the hair promoting effect of RA, we believe that its antiinflammatory and anti-microbial activity contribute to protection of folliculitis. The antiinflammatory properties are thought to be based on the inhibition of lipoxygenases and cyclooxygenases and the interference of RA with the complement cascade [52]. In addition, there was a recent report that RA in *Perilla frutescens* demonstrated effective hair growth regeneration potential [53].

Tannins, commonly referred to as tannic acid, are water-soluble polyphenols that are present in varying concentrations in plant foods and in relatively high concentrations in red wines and teas [54]. Tannins have been reported to exert anticarcinogenic, antimutagenic, antimicrobial and astringent activities [54–56]. As described above, EGCG in green tea exhibits a potential action for the prevention or treatment of alopecia by inhibiting 5*α*-reductase [31–33]. Similarly, tannin was reported to have 5*α*-reductase inhibitory effect [57]. We think the hair promoting effect with tannin of green tea in present study is related to the inhibition of 5*α*-reductase.

In addition, when compared with the finasteride group, the experimental group (both the 200 and 400 mg/kg groups) showed higher levels of expression of IGF-1, although this difference was not statistically significant. Similarly, the experimental group (400 mg/kg group) showed lower levels of TGF-*β*1 expression than did the finasteride group, although this was also not statistically significant.

Throughout the 25-day period of observation, no fatalities occurred in any of the mice group. All mice appeared to be active and well-groomed before euthanasia. No significant weight loss was noted (Additional file 1).

The limitations of the present study include the small number of animals and the absence of data on topical application of this medication, which is currently under investigation in our laboratory. HPLC–UV chromatogram in current study showed that quercetin, RA and tannin were contained in HC, PFVA, and GT, respectively (Fig. 1). However, we cannot rule out the possibility that other components in the herbal complex exert hair promoting activity. Further phytochemical analysis will be essential.

Nevertheless, the present study provides adequate preliminary data to establish a mechanism of action for this herbal complex with respect to hair regeneration. Here, we hypothesize that the multi-herbal formulation achieves its bioactivity with possible synergistic effects from each component: (1) most importantly, induction of anagen hair regrowth (modulation of IGF-1 and TGF-*β*1) by HC, (2) anti-inflammatory and anti-microbial actions [28, 29] (possible protection of folliculitis) by PFVA, and (3) inhibition of 5*α*-reductase by GT [31, 33].

## Conclusions

In present study, we studied a tri-mix herbal complex (HC, PFVA, and GT), which is commercially available and popularly used in Korea, despite the lack of reasonable experimental evidence of its effectiveness for hair regeneration. Overall, based on our analysis of gross morphology, histological finding, and expression of IGF-1 and TGF-*β*1 in a C57BL/6 mice model, our results supported the hair regeneration effect of this herbal complex.

## Additional files

Additional file 1: Table S1. Change in body weight (g) of C57BL/6 mice during study period. **Table S2.** Raw data of body weight (g) of C57BL/6 mice. **Table S3.** Raw data of gross area of hair regrowth (%) at day 25. **Table S4.** Raw data of diameter (μm) of hair follicle. **Table S5.** Raw data of length (μm) of hair follicle. **Table S6.** Raw data of distance (μm) between hair bulb and dermis. **Table S7.** Raw data of number of hair follicles in deep subcutis. **Table S8.** Raw data of western blot assay (IGF-1). **Table S9.** Raw data of western blot assay (TGF-*β*1) (ZIP 77 kb)

## Abbreviations

ANOVA: One-way analysis of variance
EGCG: Epigallocatechin-3-gallate
GT: Green tea
HC: *Houttuynia cordata* Thunb
HPLC: High performance liquid chromatography
HS: Hair shaft
IGF: Insulin-like growth factor
PFVA: *Perilla frutescens* Britton *var. acuta*
RA: Rosmarinic acid
SD: Standard deviation
SG: Sebaceous gland
TGF: Transforming growth factor
