# Supplementary material for: An Asian traditional herbal complex containing Houttuynia cordata Thunb, Perilla frutescens Var. acuta and green tea stimulates hair growth in mice
Source: BMC Complement Altern Med. 2017 Dec 2;17:515. doi: 10.1186/s12906-017-2003-x (PMC5712091; doi:10.1186/s12906-017-2003-x)
Supplement: Supplementary file 1 — Change in body weight (g) of C57BL/6 mice during study period. Table S2. Raw data of body weight (g) of C57BL/6 mice. Table S3. Raw data of gross area of hair regrowth (%) at day 25. Table S4. Raw data of diameter (μm) of hair follicle. Table S5. Raw data of length (μm) of hair follicle. Table S6. Raw data of distance (μm) between hair bulb and dermis. Table S7. Raw data of number of hair follicles in deep subcutis. Table S8. Raw data of western blot assay (IGF-1). Table S9. Raw data of western blot assay (TGF-β1) (ZIP 77 kb) [file 12906_2017_2003_MOESM1_ESM.zip › Table S1R3.doc]

**Table S1.** Change in body weight (g) of C57BL/6 mice during study period.

0 day	7 day	14 day	21 day

Mean (**±**SD)

Negative control	16.52 (±1.71)	17.60 (±1.58)	18.98 (±2.08)	20.40 (±1.66)

Finasteride	16.64 (**±**1.20)	18.04 (**±**1.15)	19.32 (**±**0.70)	19.80 (**±**0.32)

Herbal complex (200 mg/kg)	16.58 (**±**1.59)	17.90 (**±**1.19)	19.02 (**±**1.22)	20.02 (**±**1.02)

Herbal complex (400 mg/kg)	16.60 (**±**0.90)	18.10 (**±**0.71)	19.44 (**±**1.16)	20.14 (**±**1.27)

SD: standard deviation
